# Rheumatic Disease: Protease-Activated Receptor-2 in Synovial Joint Pathobiology

**DOI:** 10.3389/fendo.2018.00257

**Published:** 2018-05-23

**Authors:** Kendal McCulloch, Sarah McGrath, Carmen Huesa, Lynette Dunning, Gary Litherland, Anne Crilly, Leif Hultin, William R. Ferrell, John C. Lockhart, Carl S. Goodyear

**Affiliations:** ^1^Institute of Biomedical & Environmental Health Research, University of the West of Scotland, Paisley, United Kingdom; ^2^Institute of Immunity, Infection & Inflammation, University of Glasgow, Glasgow, United Kingdom; ^3^Respiratory, Inflammation and Autoimmunity, Innovative Medicines and Early Development, AstraZeneca, Mölndal, Sweden

**Keywords:** protease-activated receptor-2, rheumatoid arthritis, osteoarthritis, bone, cartilage, synovium

## Abstract

Protease-activated receptor-2 (PAR2) is one member of a small family of transmembrane, G-protein-coupled receptors. These receptors are activated *via* cleavage of their N terminus by serine proteases (e.g., tryptase), unveiling an N terminus tethered ligand which binds to the second extracellular loop of the receptor. Increasing evidence has emerged identifying key pathophysiological roles for PAR2 in both rheumatoid arthritis (RA) and osteoarthritis (OA). Importantly, this includes both pro-inflammatory and destructive roles. For example, in murine models of RA, the associated synovitis, cartilage degradation, and subsequent bone erosion are all significantly reduced in the absence of PAR2. Similarly, in experimental models of OA, PAR2 disruption confers protection against cartilage degradation, subchondral bone osteosclerosis, and osteophyte formation. This review focuses on the role of PAR2 in rheumatic disease and its potential as an important therapeutic target for treating pain and joint degradation.

## Protease-Activated Receptor-2 (PAR2)

Protease-activated receptors (PARs) 1–4 are a family of transmembrane, G-protein-coupled receptors. These receptors lack a conventional soluble ligand. Instead they are activated by serine protease-mediated cleavage of their N terminus, unveiling a “tethered ligand” which can then bind the second extracellular loop. Once activated, the continued interaction between the receptor and its tethered ligand means it cannot be subsequently reactivated. Instead, PARs are internalized and degraded post-activation. PARs 1, 3, and 4 are primarily cleaved by thrombin (1) and play important roles in vascular physiology, the coagulation pathway and the immune system. PAR2, in contrast, is activated by a number of serine proteases such as trypsin (2), mast cell tryptase (3), neutrophil proteinase 3 (4), and matriptase (5), many of which are generated and released during tissue injury and/or inflammation. In the last decade a strong link has emerged between PAR2 and the innate and adaptive immune responses (6), and the stromal compartment. Furthermore, given the previously established role for PAR2 in nociception (7), there is now substantial evidence to support the therapeutic targeting of this receptor to combat pain as well as inflammation and joint destruction and in the rheumatic diseases. Notably, while there are no specific and potent PAR2 antagonists in clinical trials, a recent publication of the crystal structure of PAR2 (8) will accelerate design and availability of such compounds for future trials in various diseases.

## Rheumatoid Arthritis (RA)

**Figure 1:**
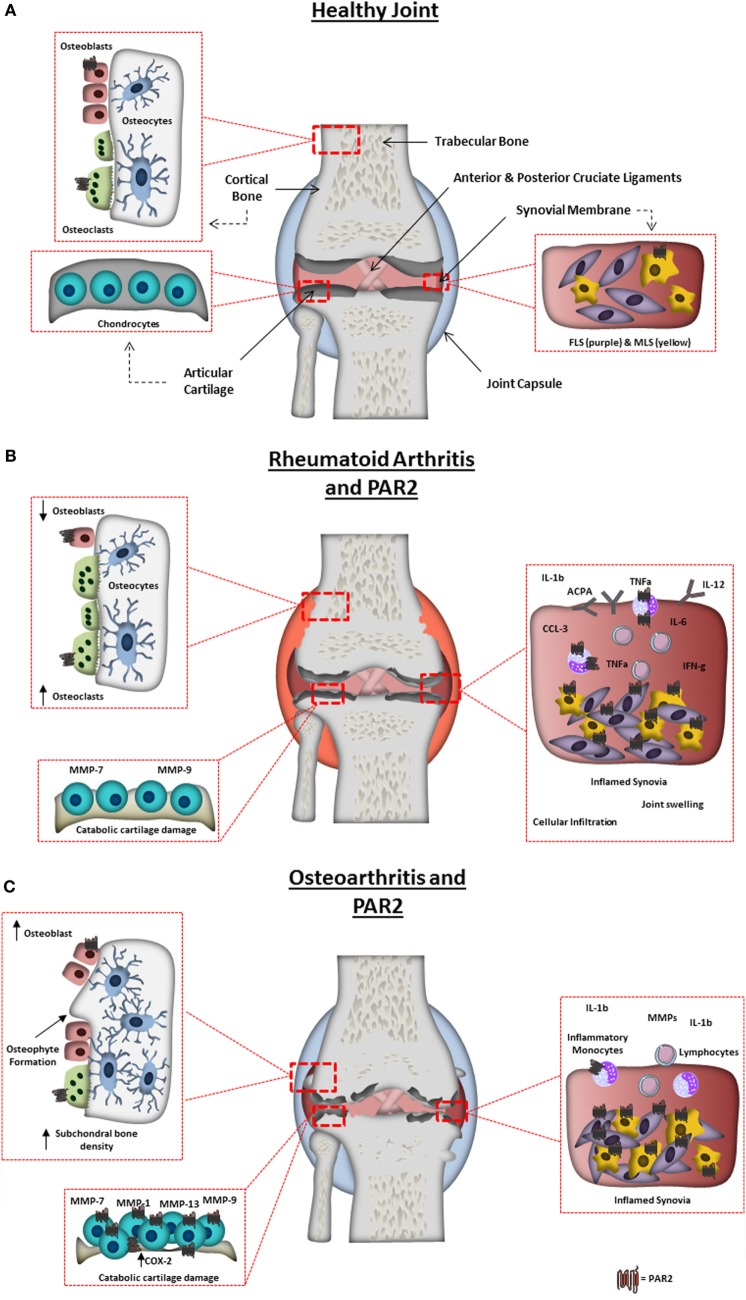
Arthritic joint changes and the pathogenic role of protease-activated receptor-2 (PAR2). **(A)** Schematic representation of a normal healthy joint, highlighting the various joint tissues, cell types residing within, and the expression of PAR2 in the healthy joint. **(B)** A schematic representation of a rheumatoid arthritis-affected joint, highlighting characteristic changes including: synovitis, degradation of the articular cartilage; bone erosion and increased inflammatory factors. PAR2 expression is also highlighted in pathogenic areas known to be influenced by PAR2 from animal studies or *in vitro* cell work. Including, increase in PAR2 expression in monocytes, macrophages, and fibroblast-like synoviocytes, and contributing to monocyte IL-6 production, macrophage cytokine production, autoantibody production, bone erosion, and cartilage destruction. **(C)** Schematic representation of an osteoarthritis (OA)-affected joint, highlighting characteristic changes including: synovitis; degradation of the articular cartilage; osteophyte formation; subchondral bone sclerosis and pannus formation. PAR2 expression is also highlighted in cells/tissues and pathogenic outcomes known to be influenced by PAR2 from animal studies or *in vitro* cell work. Increased levels of PAR2 have been found in OA chondrocytes, fibroblasts, and macrophages, with PAR2 known to play a role in osteophyte formation, cartilage degradation (through catabolic protease production), and inflammation.

The arthritides are a heterogeneous group of joint diseases with the common endpoint of structural joint degradation; the most overtly inflammatory of these is RA. This chronic, autoimmune disorder affects an estimated 1% of the world population (9). It is characterized by swelling, pain, and loss of mobility, and manifests as a result of a break in immune tolerance toward antigens associated with articular joints in genetically susceptible individuals. This leads to an adaptive driven immune response to self-antigens, such as IgG (rheumatoid factor) or posttranslationally modified proteins (citrullinated protein). The RA inflammatory synovial infiltrate consists of auto-reactive B and T cells, inflammatory monocytes, and mast cells, which together produce a network of pro-inflammatory cytokines (e.g., TNF-α and IL-6) (10), chemokines (e.g., CCL2 and CXCL8), and proteases, contributing to a hypoxic and inflammatory environment. The synovial membrane expands due to reduced apoptosis, increased inflammatory mediators (e.g., IL-6), and catabolic protease production by fibroblast-like synoviocytes (FLS). This aggressive phenotype is maintained (11) through epigenetic imprinting, creating an almost “transformed” synovial membrane (12). FLS hyperplasia creates structural damage due to thickening of the synovial membrane, creating an invasive pannus that can erode neighboring tissue. Structural damage is also caused by the immune cell infiltrates invading the juxta-articular bone and calcified cartilage, led by excessive osteoclast activity (derived from infiltrating myeloid cells, e.g., monocytes) and TNF-α-driven mechanisms (13). Pathogenic mechanisms driving structural joint changes in RA are summarized in Figure 1.

### Synovium

The first “proof of concept” that PAR2 has a direct role in chronic inflammatory arthritis was demonstrated by Ferrell et al. using an adjuvant-induced monoarthritis model in wild-type (WT), PAR2 deficient homozygote (*Par2^−/−^*) and PAR2 heterozygote (*Par2^+/−^*) mice. This innate immune-mediated arthritis model revealed almost complete ablation of synovitis in *Par2^−/−^* compared with WT mice, with heterozygotes demonstrating an intermediate phenotype (14). The immunological role of PAR2 in murine RA models was further investigated *in vivo* using the gold standard model of inflammatory RA, collagen-induced arthritis (CIA) in both DBA/1 and C57Bl/6J mice. Arthritis was significantly reduced following therapeutic treatment with PAR2 inhibitors (both small-molecule antagonist ENMD-1068 and SAM-11 monoclonal antibody) (15). This was associated with an altered immune response in secondary lymphoid tissue, whereby PAR2 inhibition significantly reduced IL-17 and IFN-γ levels and had a minor impact on TNF-α, IL-1β, IL-6, IL-12, CCL3, and GM-CSF expression (15). Furthermore, anti-type II collagen antibodies were also significantly reduced after PAR2 inhibition. This compelling evidence supports the immunological role of PAR2 in inflammatory joint disease, pinpointing reductions in key synovitis-associated cytokines, and links PAR2 to the induction of adaptive antibody responses. The upstream ability of PAR2 to modulate multiple cytokine pathways potentially represents an over-arching approach to target multiple immunopathological pathways. It is therefore interesting to speculate that PAR2-mediating therapeutic strategies could provide an alternative to those currently focused on targeting these pathways (i.e., JAK inhibitors).

Subsequent translational studies implicated a pathogenic role for PAR2 in the context of human RA. Notably, the level of PAR2 transcript and protein was significantly increased in both synovial tissue biopsies and isolated FLS from RA synovium when compared with osteoarthritis (OA) patients (16). An inflammatory role for PAR2 in the synovia was further implicated by the correlation of PAR2 expression in RA patients with the extent of synovial pathology (16). To understand the underlying mechanism, studies have been conducted to dissect the pathways influenced by PAR2 activation in cells associated with RA synovial infiltrates. Crilly and colleagues investigated the cell surface expression of PAR2 on CD14^+^ circulating monocytes (which likely migrate into the inflamed joint and differentiate to macrophages or osteoclasts) during both RA remission and flare (17). These studies demonstrated that the expression of PAR2 on patient monocytes correlated with classic biomarkers of disease flare such as erythrocyte sedimentation rate and C reactive protein levels. Importantly, the elevated levels of PAR2 surface expression in CD14^+^ monocytes was significantly reduced in patients receiving conventional DMARDs (17, 18). This direct correlation between receptor expression and disease activity supports a role for PAR2 in driving inflammatory disease. Moreover, at a functional level, the activation of PAR2 on human monocyte *in vitro* derived macrophages, *via* the agonist peptide SLIGKV, fundamentally altered the cellular cytoskeleton (elongated spindle-like appearance) and enhanced TNF-α production, both in the presence or absence of LPS stimulation (19). This confirms that enhanced PAR2 expression on monocytes (17) translates to a pro-inflammatory phenotype. However, the potential differential expression of PAR2 or the functional role of the receptor in different monocyte subpopulations remains to be elucidated. Thus far the PAR2 expression on RA monocyte subsets has been limited to analysis of CD14^+^ monocytes. Interrogation of PAR2 expression in the full monocyte compartment in RA, including classical monocytes (CD14^++^CD16^−^, responsible for higher cytokine production and inflammation), intermediate monocytes (CD14^++^CD16^+^) and non-classical monocytes (CD14^+^CD16^++^, patrolling) may provide further insights into the inflammatory role of this receptor (20).

In summation, overall increased PAR2 expression is seen across multiple cell types of the RA synovium. Assessment of PAR2 activity both *in vivo* and *in vitro* indicates that this pathway may at least in part be responsible for the disease associated activity of these cell types. Therefore, PAR2 may be dysregulated in cells of the innate immune system and stromal FLS and thus represent a therapeutically tractable pathway in RA.

### Cartilage

The main contributor to cartilage degradation in RA is a hyperplastic synovium (21). The protein-binding properties of cartilage becomes altered due to decreased lubricin expression and loss of other protective mechanisms within the synovium (22). This leads to FLS adhesion and invasion of the bone and cartilage, resulting in the formation of pannus. Metalloproteinases (MMPs) are released from the chondrocytes and synovium, contributing to subsequent cartilage degradation. FLS predominantly release MMP-14 that also degrades the cartilage matrix (23). Chondrocytes also express a disintegrin and MMP with thrombospondin motifs 5 (ADAMTS 5), which has the ability to degrade aggrecan (ECM component) (24), as well as MMP-1; the major collagenase associated with RA (25). Moreover, the secretion of IL-1β within the synovial environment, which drives catabolic activity within chondrocytes, also upregulates PAR2 on chondrocytes and can in turn be regulated by PAR2 activation (16).

Using the adjuvant-induced arthritis model to mimic chronic inflammatory arthritis in mice, the absence of PAR2 conferred protection against cartilage damage. By comparison, cartilage was entirely replaced by pannus in WT mice, with associated erosion of the cortical bone, while *Par2^+/−^* mice exhibited an intermediate level of protection against cartilage damage (14). The ability of PAR2 to regulate adaptive immune pathways potentially responsible for driving cartilage damage in RA was explored in the CIA model. Histological analysis of the paws revealed that *Par2^−/−^* mice had significantly lower cartilage damage than WT controls (15), suggesting that this protection observed in *Par2^−/−^* mice was related to the capacity of PAR2 to drive the release of cytokines, including IL-1β, known to induce degradation of cartilage and ECM components in this inflammatory arthritis model. While PAR2 drives cartilage damage in inflammatory arthritis, the pathogenic mechanism is poorly understood. Unanswered questions include whether PAR2 can modulate the erosive environment leading to cartilage damage, or whether its activation undermines chondrocyte biology/viability. Moreover, translational studies are required to determine if this pathogenic role is relevant in human RA.

### Bone

Dysregulation of the homeostatic bone remodeling process is recognized to be a key feature of RA. This is associated with localized bone erosion at the site of inflammation, and systemic bone loss, that can contribute to an increased risk of fracture (26, 27). The high prevalence of synovial pro-inflammatory factors promotes osteoclastogenic activity resulting in focal bone erosions, especially at the interface of bone and pannus (28). In addition, the balance of bone formation and resorption is further perturbed in these patients because the inflammatory environment, including IL-1β and IL-6 inhibit the migration and activity of the bone forming osteoblasts (29, 30). The role of PAR2 in bone erosion in inflammatory disease remains unclear, and current literature regarding PAR2 in osteoclasts is conflicting. Studies have shown PAR2 is expressed in osteoblasts (31) and CD14^+^ monocytes (17), the latter being osteoclast precursors. Another study explored PAR2-mediated bone functions, in a bone marrow-derived coculture system; addition of soluble factors resulted in osteoblast differentiation, subsequently promoting osteoclastogenesis. When PAR2 activating peptides were included in this system, the number of tartrate-resistant acid phosphatase (TRAP) positive osteoclasts decreased. However, when osteoclasts were cultured alone (without osteoblasts), PAR2 activation had no effect. This suggests PAR2 activation inhibited osteoclastogenesis *in vitro via* an osteoblast-mediated mechanism, and it was proposed that this mechanism may protect bone from uncontrolled resorption (32). In contrast, a study of skeletal development in *Par2^−/−^* mice revealed an increase in total bone volume and a deficit in the area of bone associated with osteoclasts. Moreover, *in vitro* osteoclastogenesis assays demonstrated that osteoclasts derived from precursors lacking PAR2 had reduced levels of TRAP (33). While conflicting, these studies do indicate that PAR2 could play a fundamental role in the differentiation of bone precursor cells into their mature forms, and therefore bone remodeling in RA may be influenced *via* PAR2 modulation.

## Osteoarthritis

As the most prevalent musculoskeletal disease globally (34), OA is a chronic debilitating condition affecting an estimated 100 million Europeans. It has a dramatic impact on quality of life (35), through associated pain, swelling of the joint and lack of mobility/loss of normal joint function. In the absence of effective disease modifying therapies, analgesia, and ultimately arthroplasty remain the only real options for most patients. In the UK, between 2007 and 2008 over 140,000 primary hip and knee replacements were performed, with an addition of more than 10,000 revision arthroplasties. This represents an NHS cost burden in excess of £1 billion per year (36). Risk factors linked to OA include gender, obesity, genetic factors, joint injury and advancing age, with approximately 80% of the population >65 years old showing radiological evidence of OA (37). Depending on the stage of disease, an OA joint can exhibit various characteristics such as degradation of the articular cartilage, osteophyte formation (bony outgrowths), subchondral bone sclerosis, pannus formation, and inflammation of the synovial membrane (synovitis) (Figure 1C). OA is inherently heterogeneous: there is substantial inter-patient variability in clinical features, biochemical characteristics and treatment responses. This, together with the subtle onset of OA and lack of definitive biomarkers, makes early diagnosis extremely challenging. What was once considered a degenerative “wear and tear” disease is now recognized as a complex disorder involving various molecular pathways and inflammatory mediators (38). The pathogenic mechanisms of this complex disease are still not fully understood, and accordingly this unmet clinical need is a major focus of research.

### Synovium

Unlike RA, there are no systemic signs of inflammation or evidence of neutrophils within OA synovial fluid. There is, however, evidence of inflammatory (B and T cell) infiltrate into the synovium (39), together with increased levels of pro-inflammatory cytokines (IL-1β and TNF-α) and catabolic proteases (e.g., MMPs). These are thought to be instrumental in chondrocyte mediated cartilage destruction.

Scoring of synovial thickness and monocyte infiltrates in OA demonstrated that OA synovitis correlates with levels of PAR2 expression in synovial tissue (40). While synovitis scores were reduced in OA compared with RA, increased PAR2 levels were directly associated with inflammatory conditions across all patient groups. These studies also demonstrated that a PAR2 antagonist, ENMD-1068, dose-dependently inhibited TNF-α production from OA synovial explant cultures. Increased PAR2 expression in OA synovia would therefore appear to be not merely a passive marker of inflammation, but an active contributor to pro-inflammatory cytokine production.

### Cartilage

Chondrocytes are the primary source of enzymes such as MMPs responsible for the metabolism of the cartilage matrix in OA (41). Pro-inflammatory cytokines synthesized and released by both chondrocytes and the synovial membrane are important in the development and progression of OA as they play a central role in inducing cartilage catabolic processes (42). MMPs are considered crucial in cartilage catabolism, as they collectively have the ability to degrade all components of the ECM (43).

Protease-activated receptor-2 transcript is sevenfold higher in chondrocytes isolated from the cartilage of OA patients compared with control femoral fracture patients who have no evidence of arthropathy (44). Moreover, *in vitro* culture and subsequent passage of chondrocytes demonstrated that only in OA-derived samples is there maintenance of PAR2 protein. This indicates potential epigenetic alterations in these cells enabling them to retain PAR2 expression (44).

Selective activation of PAR2 in chondrocytes mediates a stress-activated protein kinases (SAPK)/p38 and extracellular signal-regulated kinase 1/2 signaling cascade, which results in the generation of MMP-1, MMP-13 and cyclooxygenase 2 (45). In OA and RA, MMP-1 and -13 and ADAMTS aggrecanases are strongly associated with the degradation of collagen and aggrecan (46), the main components of the ECM. Moreover, pro-inflammatory cytokines such as IL-1β and TNFα have been shown to upregulate PAR2 expression in OA chondrocytes (44), and PAR2 appears to regulate the synovial release of IL-1β, a chondrocyte catabolin (16, 47). These findings collectively implicate a potential auto-feedback loop between PAR2, inflammation and cartilage destruction.

In the murine destabilization of the medial meniscus (DMM) model, PAR2 has been identified as a critical checkpoint in the pathogenesis of experimental OA (48). *Par2^−/−^* mice exhibit substantially less cartilage damage at 4 weeks’ post DMM than WT littermates (48), a key finding confirmed in a later study (49), and across several groups (50, 51). Notably, 8 weeks’ post DMM, *Par2^−/−^* mice retain cartilage integrity while WT mice have more pronounced joint destruction (48). Using either a PAR2 agonist or monoclonal antibody specifically targeting the PAR2 protease cleavage site, inhibition of PAR2 in WT mice was observed to be as equally effective as gene deletion in curtailing OA progression *in vivo* (48), but only in early (1 week) stages of disease.

To further test the hypothesis that PAR2 drives cartilage damage in the DMM model (48, 50, 51), additional experiments using a viral vector containing human PAR2 (h*Par*2) showed that intraarticular injection of h*Par2* in *Par2^−/−^* mice restored the pathogenic phenotype (49). While Jackson et al. demonstrated a significant correlation between cartilage damage and subchondral bone sclerosis (51), Huesa et al. demonstrated cartilage damage but no significant difference in subchondral bone sclerosis in the hPAR2-transfected *Par*2*^−/−^* mice compared with vector controls (49). The authors concluded that, in the DMM model, even in the absence of subchondral bone sclerosis, cartilage damage is mediated *via* PAR2. However, it should be appreciated that subtle differences in PAR2-dependent pathological features in these studies may reflect divergence of methodologies or temporal analyses employed by these groups. In summation, PAR2 activity, through the stimulation of MMPs and various other proteases appears to promote cartilage degradation. PAR2 deficiency may confer protection against cartilage erosion *via* inhibition of MMP production and/or alteration of chondrocyte phenotype. Many questions remain with regard to the role of PAR2 in chondrocyte biology and how it affects OA etiology. Thus future studies should consider using cartilage-specific *Par2^−/−^* animals.

### Bone

Bone pathology in OA differs from RA. In addition to bone loss in the metaphysis, OA is characterized by osteosclerosis (hardening and increased density of subchondral bone) (52) and formation of osteophytes along the joint margins (53).

Protease-activated receptor-2 has recently been reported to play an essential role in osteophyte formation during DMM progression: although osteophytes are detectable in both WT and *Par2^−/−^* mice, the quantity, size, and growth of osteophytes over time is significantly reduced in *Par2^−/−^* animals. The OA phenotype was subsequently “rescued” *via* intraarticular *Par2* adenovirus transfection (49), which restored the pathogenic phenotype observed in WT animals. This appears to contradict a previous study which found no significant gross difference in osteophyte formation between WT and *Par2^−/−^* (51). However, the difference in techniques adopted in these studies (microcomputed tomography versus histology, respectively) may explain the conflicting results as the former technique offers far more quantitative measurement of osteophyte changes such as volume, density, and arboreal structure. While these studies provide important “proof of concept” that PAR2 modulates the pathogenic bone phenotype found in murine OA models, *in vitro* studies on human osteoclasts and stroma are still needed to dissect the mechanisms involved, as well as translating these findings to human OA. This is an important future direction, since PAR2 inhibition may offer protection against osteophytogenesis in OA patients.

## Conclusion

Arthritis studies have identified the cell types expressing PAR2, shown the receptor is up regulated and provided proof of concept that PAR2 inhibition offers therapeutic protection. PAR2 may therefore present a complete novel target that merits research focus since there remains an urgent need for targeted disease modifying agents in both RA and OA. Recent advances in RA biological treatments against specific cytokines and immune cells have not fulfilled their early promise, are expensive, and require an injection-based delivery system. In OA, disease modifying agents are virtually non-existent and current treatment options are severely limited.

Continuing research is required to achieve the ultimate therapeutic ambition of identifying new targets with high disease specificity and good accessibility by manageable drug delivery. While PAR2 has been recognized as a potential target with significant therapeutic value, advance in this area has been severely limited by the current lack of effective small-molecule PAR2 antagonists. Given the burgeoning problem of arthritis with an ever aging population, the recent and timely publication of the crystal structure of PAR2 (8) presents a landmark step in progressing pharmaceutical research by accelerating development of such agents for future clinical studies.

## Author Contributions

The joint first authors (KM and SM) prepared the first draft of the review, and this was then revised and finalized with input from all authors (CH, LD, GL, AC, LH, WF, JL, and CG).

## Conflict of Interest Statement

CG has received consulting fees from AstraZeneca (more than $10,000). LH is an employee of AstraZeneca. All other authors declare no conflict of interest.
